# Optimization of Extraction Conditions of Phytochemical Compounds and Anti-Gout Activity of *Euphorbia hirta* L. (Ara Tanah) Using Response Surface Methodology and Liquid Chromatography-Mass Spectrometry (LC-MS) Analysis

**DOI:** 10.1155/2020/4501261

**Published:** 2020-01-24

**Authors:** Fazleen Izzany Abu Bakar, Mohd Fadzelly Abu Bakar, Norazlin Abdullah, Susi Endrini, Sri Fatmawati

**Affiliations:** ^1^Centre of Research for Sustainable Uses of Natural Resources (CoR-SUNR), Faculty of Applied Sciences and Technology, Universiti Tun Hussein Onn Malaysia (UTHM), 84600 Muar, Johor, Malaysia; ^2^Faculty of Medicine, Universitas Abdurrab, Pekanbaru 28292, Riau, Indonesia; ^3^Department of Chemistry, Faculty of Science, Institut Teknologi Sepuluh Nopember, Surabaya, Indonesia

## Abstract

Gout is a common disease affected most of the people due to the elevation of uric acid in the blood. Flavonoid and phenolic compounds are reported to exert the anti-gout activity of medicinal plants. Hence, this study aimed at optimizing the extraction conditions of phenolic and flavonoid compounds as well as the anti-gout (xanthine oxidase inhibitory activity) *in vitro* of *Euphorbia hirta* using response surface methodology (RSM). The plant part used was the whole plant excluding roots. The effects of three independent variables (extraction time, *X*_1_; extraction temperature, *X*_2_; and solid-to-liquid ratio, *X*_3_) on three response variables (total flavonoid content, *Y*_1_; total phenolic content, *Y*_2_; and xanthine oxidase inhibitory activity, *Y*_3_) were determined using central composite design (CCD) while phytochemical profiling of the extracts was determined by liquid chromatography-mass spectrometry (LC-MS). Quadratic models produced a satisfactory fitting of the experimental data with regard to total flavonoid content (*r*^2^ = 0.9407, *p* < 0.0001), total phenolic content (*r*^2^ = 0.9383, *p* < 0.0001), and xanthine oxidase inhibitory activity (*r*^2^ = 0.9794, *p* < 0.0001). The best extraction conditions observed for total flavonoid content, total phenolic content, and xanthine oxidase inhibitory activity were at a temperature of 79.07°C for 17.42 min with solid-to-liquid ratio of 1 : 20 g/ml. The optimum values for total flavonoid, total phenolic, and xanthine oxidase inhibitory activity were 67.56 mg RE/g, 155.21 mg GAE/g, and 91.42%, respectively. The main phytochemical compounds in the optimized *E*. *hirta* extract are neochlorogenic acid, quercetin-3*β*-D-glucoside, syringic acid, caffeic acid, ellagic acid, astragalin, afzelin, and quercetin. As conclusion, this study clearly demonstrated the best conditions to obtain higher xanthine oxidase inhibitory activity and phytochemical compounds which can be further used for the development of anti-gout agents.

## 1. Introduction

*Euphorbia hirta* L. or locally known as Ara tanah or Gelang susu in Malaysia is from the family of Euphorbiaceae. The genus of *Euphorbia* is considered to be the largest in the Euphorbiaceae family with about 1600 species and it is native to tropical and subtropical regions of Asia, Africa, and Central as well as South America [1]. This plant was found to possess potent antimicrobial [2], antidiarrhoeic [3], antihyperglycemic [4], anti-inflammatory [5], and anticancer [6] activities. In fact, in Malay traditional medicine, it has been used widely to treat various diseases including skin problems, gastrointestinal disorders, diarrhoea, and ulcer [2]. However, in Ayurvedic treatment, this plant has been used traditionally to treat worm infestations in children, dysentery, jaundice, pimples, digestive problems, female disorders, respiratory ailments (cough, coryza, bronchitis, and asthma), and tumors [1]. In addition, the decoction or infusion of this plant has been used widely by practitioners of traditional medicine in treating asthma, kidney stones, heartburn, diarrhea, coughs, and menstrual problems [7]. Interestingly, many phytochemical compounds such as saponin, sterol, terpene, alkaloids, polyphenols, tannins, terpenoids, steroids, and flavonoids have been found in the aerial parts of this plant [4, 7]. This plant is also rich in minerals such as zinc, potassium, calcium, and magnesium [4]. In 2007, three compounds from the methanolic extract of *E*. *hirta*, namely, afzelin, quercetin, and myricetin which showed potent inhibition against malaria have been successfully isolated [8].

Extraction is considered to be one of the important steps in discovering potential bioactive compounds from plant materials, considering their quantity and type of bioactive compounds as they are widely used in nutraceutical, pharmaceutical, and cosmetic products as well as functional food ingredients and food additives [9, 10]. The type and concentration of solvent, temperature, time, pH, solid-liquid ratios, pressure, and the particle size of the plant have been identified from the previous studies as important factors affecting the extraction efficiency of the plant bioactive compounds [11–13]. Traditional method, which is known as the one-factor-at-a-time, has been used widely by many researchers in optimizing specific parameters in which only one factor plays a role as a variable at a time while keeping all other factors constant [14]. However, this method is considered to be less reliable as it does not include interactive effects among factors, time-consuming, and expensive. Misleading conclusions and false conditions also might be occurred at the end of this experiment method, ignoring the true significant factors during the extraction process [9]. To overcome this problem, response surface methodology (RSM) has been introduced and widely used nowadays for optimizing the extraction conditions. RSM is considered to be a powerful mathematical technique used widely in many industries for technological operations in order to optimize certain experimental conditions. Moreover, it evaluates the effects between the multiple factors and their interactions towards one or more response variables simultaneously, producing lesser number of experimental measurements [15]. It also helps to locate the region where the extraction is optimized by using clearer visual aid [16]. Based on the previous literature, the phenolic and flavonoid contents of guava puree [17], *Curcuma zedoaria* leaves [16], apples [18], *Feronia limonia* fruit [15], and *Mangifera pajang* [9] have been successfully optimized using RSM.

Xanthine oxidase inhibitory activity (XO) is an enzyme assay used widely in determining the anti-gout activity of the plant extracts. XO is known as the main enzyme causing the accumulation of uric acid in the blood which eventually leads to gout. The mechanism involves where it catalyses the oxidation of hypoxanthine into xanthine and then uric acid [19]. Optimization of extraction method to obtain higher total flavonoids and phenolics as well as XO inhibitory activity from *E*. *hirta* is essential for future nutraceutical product development especially for the gout treatment. To the best of our knowledge, this is the first report describing optimization of extraction process of phytochemical compounds and XO inhibitory activity from *E*. *hirta*. Therefore, this study aimed at optimizing the extraction temperature, time, and solid-to-liquid ratio for the extraction of total flavonoid content, total phenolic content, and anti-gout activity from *E*. *hirta* whole plant excluding roots using RSM.

## 2. Materials and Methods

### 2.1. Chemicals and Reagents

Gallic acid, xanthine, xanthine oxidase (buttermilk), and potassium dihydrogen phosphate (KH_2_PO_4_) were purchased from Sigma-Aldrich Chemicals (St. Louis, MO, USA). Folin–Ciocalteu reagents were obtained from Merck (Germany). Allopurinol, rutin, aluminum chloride hexahydrate, sodium nitrite, sodium hydroxide, and dipotassium hydrogen phosphate (K_2_HPO_4_) were of the highest purity (95–99%).

### 2.2. Plant Materials

The plant part used was the whole plant excluding roots. *E*. *hirta* was collected from the Department of Agriculture, Serdang, Selangor, Malaysia, and authenticated by Dr. Mohd Firdaus Bin Ismail (Institute of Bioscience, Universiti Putra Malaysia), and the voucher specimen was deposited in the Herbarium of the same institute under the number MFI0084/19.

### 2.3. Sample Extraction

The whole plant excluding root was soaked in water, washed, and oven-dried at 40°C for 72 hours. Then, it was ground and stored in a −20°C freezer prior to the extraction process. In order to mimic the human consumption, the plant was prepared using hot boiling water extraction procedure whereby each 1.0 g of dried plant was infused in 10–20 ml of distilled water at temperature between 50 and 100°C and continuously stirred for 5–60 min (Table 1) using a magnetic stirrer under 300 rpm. The infusion was left to cool to 15 min. Then, the extract was filtered using Whatman No. 1 filter paper and subjected to the xanthine oxidase inhibitory activity assay spectrophotometrically at 295 nm, for determination of total flavonoid and total phenolic contents.

### 2.4. Experimental Design

Central composite design (CCD) was developed in order to obtain optimized extraction conditions for higher total flavonoid content, total phenolic content, and xanthine oxidase inhibitory activity of *E*. *hirta*. CCD was chosen in this study as it provides more design points on each variable. Three selected independent variables in this study were extraction time (*X*_1_: 5–60 minutes), extraction temperature (*X*_2_: 50–100°C), and solid-to-liquid ratio (*X*_3_: 1 : 10–1 : 20 g/ml), while the dependent variables chosen were total flavonoid content (*Y*_1_), total phenolic content (*Y*_2_), and xanthine oxidase inhibitory activity (*Y*_3_). The responses of total flavonoid content and total phenolic content were critical to be studied as the presence of both of them in the plants have been reported to be the reasons for treating the gout disease while xanthine oxidase inhibitory activity was the *in vitro* enzyme assay which has been used widely for determining the antigout activity of the plants. The complete design was generated by a statistical software package (Design Expert, version 7.0, Minneapolis, USA) which consists of 20 combinations including six replicates at the central point (Table 1).

### 2.5. Total Flavonoid Content (TFC) Determination

TFC of the plant extract was determined using aluminium chloride method [20]. The absorbance of the mixture was measured spectrophotometrically at 510 nm, and the results obtained were expressed as milligram rutin equivalent per gram (mg RE/g) based on the standard curve of various concentrations of rutin (0–100 *μ*g/ml).

### 2.6. Total Phenolic Content (TPC) Determination

TPC of the plant extract was determined using Folin–Ciocalteu's colorimetric method with slight modification [21]. The absorbance was spectrophotometrically measured at 725 nm, and the results obtained were expressed as milligram gallic acid equivalent per gram (mg GAE/g) based on the standard curve with various concentrations of gallic acid (0 − 100 *μ*g/ml).

### 2.7. Xanthine Oxidase (XO) Inhibitory Activity Assay

The inhibitory effect on XO was measured spectrophotometrically at 295 nm under aerobic condition following the existing method with some modifications [22, 23] where allopurinol (0–100 *μ*g/ml) was used as a positive control. The degree of XO inhibitory activity was calculated by using the following equation:

(1) $$\begin{array}{c} \%\text{ XO inhibition}=\left(1-\frac{\beta }{\alpha }\right)\times 100\%, \end{array}$$

where *α* is the activity of XO without test extract and *β* is the activity of XO with test extract.

### 2.8. Liquid Chromatography-Mass Spectroscopy (LC-MS) Analysis

Chromatography was performed according to Zakaria et al. [24] where 20 *μ*L of sample was injected onto a Agilent C18 reverse-phase column (4.0 × 250 mm, 1.8 *μ*m) and held at 50°C with a constant flow rate of 0.4 mL/min and total LC run time of 30 min. Sample elution was performed in a gradient manner using mobile phase comprising of water containing 0.1% acetic acid (solvent A) and HPLC grade acetonitrile containing 0.1% acetic acid (solvent B). The mobile phase composition (A : B) was gradually increased from 5.95 to 15.85 over 25 min and returned to the initial condition (95%) for 5 min for solvent A and 5% to 85% for 25 min and then decreased to the initial condition (5 : 95) over the next 5 min. For the mass analysis, the source conditions were as follows: nebulizer pressure was 40 psi, drying gas flow was set at 12 L/min, and drying gas temperature was 350°C and the MS/MS acquisitions were performed in the negative and positive electrospray ionization mode, for the mass range of 150 to 1500 m/z. Data acquisition was performed by Agilent MassHunter workstation Data Acquisition, while data processing was carried out with MassHunter Qualitative Analysis software. In addition, MS/MS experiments were carried out in the automatic and multiple reaction monitoring (MRM) mode where automatic MS/MS low-energy collision dissociation (CID) was performed at 5–8 eV collision energy. Peak identification was carried out based on comparison with literature values and online databases.

### 2.9. Statistical Analysis

Design Expert® Software (version 7, Stat-Ease Inc., Minneapolis, MN) was used in this study. A response surface analysis was employed to determine the regression coefficients and statistical significance of the model terms and to fit the mathematical models of the experimental data. The adequacy of the model was predicted through the regression analysis (*r*^2^) and the analysis of variance (ANOVA) (*p* < 0.05).

## 3. Results and Discussion

### 3.1. Fitting the Response Surface Models

Fitting the models are crucial in interpreting the accuracy of the RSM mathematical models for prediction of the TFC, TPC, and XO inhibitory activity of *E*. *hirta* extract. In this present study, the relationship between the response functions (TFC, TPC, and XO inhibitory activity) and the independent variables (extraction time, extraction temperature, and solid-to-liquid ratio) was successfully identified by CCD. The results of the responses for the 20 runs following the experimental design are shown in Table 2. The yield of the TFC ranged from 51.11 to 65.55 mg RE/g while TPC ranged from 120.34 to 152.95 mg GAE/g. In terms of XO inhibitory activity, the highest activity was 97.57% whereas the lowest was 72.96%. As suggested by the Design Expert software, a quadratic polynomial model was selected and fitted well for all three independent variables and responses. In terms of coded values, the predicted responses for the TFC, TPC, and XO inhibitory activity could be expressed by the second-order polynomial equation via multiple regression analysis:

(2) $$\begin{array}{c} Y_{\text{TFC}}=60.46+1.54X_{1}+1.11X_{2}+2.64X_{3}-2.28X_{1}X_{2}+1.28X_{1}X_{3}+1.28X_{2}X_{3}-1.72X_{1}^{2}-5.73X_{2}^{2}+2.84X_{3}^{2}, \end{array}$$

(3) $$\begin{array}{c} Y_{\text{TPC}}=142.28+2.47X_{1}+2.50X_{2}+4.84X_{3}-5.72X_{1}X_{2}+3.08X_{1}X_{3}+1.71X_{2}X_{3}-2.48X_{1}^{2}-17.24X_{2}^{2}+8.88X_{3}^{2}, \end{array}$$

(4) $$\begin{array}{c} Y_{\text{XO inhibitory activity}}=\;95.03-3.30X_{1}-1.12X_{2}-6.27X_{3}-0.63X_{1}X_{2}3.48X_{1}X_{3}-0.74X_{2}X_{3}-0.16X_{1}^{2}-2.91X_{2}^{2}-3.32X_{3}^{2}, \end{array}$$

where *X*_1_, *X*_2_, and *X*_3_ are the coded variables for extraction time, extraction temperature, and solid-to-liquid ratio, respectively. A negative sign in each equation represents an antagonistic effect of the variables, and a positive sign represents a synergistic effect of the variables [16].

Positive coefficients for *X*_1_, *X*_2_, and *X*_3_ were observed in equation (2), indicating that TFC was increased with the increase of extraction time (5–60 min), extraction temperature (50–100°C), and solid-to-liquid ratio (1 : 10–1:20). The same observation was also found for TPC where positive coefficients for *X*_1_, *X*_2_, and *X*_3_ were observed in equation (3), indicating that TPC was increased with the increase of extraction time (5–60 min), extraction temperature (50–100°C), and solid-to-liquid ratio (1 : 10–1 : 20). In contrast, negative coefficients for *X*_1_, *X*_2_, and *X*_3_ were observed in equation (4), indicating that XO inhibitory activity was decreased with the increase of extraction time (5–60 min), extraction temperature (50–100°C), and solid-to-liquid ratio (1 : 10–1 : 20). The RSM model coefficients were validated by analysis of variance (ANOVA) of the response variables for the quadratic polynomial model summarized in Supplementary Materials (Tables [Supplementary-material supplementary-material-1], [Supplementary-material supplementary-material-1], and [Supplementary-material supplementary-material-1]). For the responses of TFC, TPC, and XO inhibitory activity, the models were highly significant when the computed *F*-values were greater than the tabulated *F*-value and the probability values were low (*p* < 0.001), indicating that the individual terms in each response model were significant on the interaction effect. In addition, the coefficient of determination (*r*^2^) of the models was 0.9407, 0.9383, and 0.9794, respectively, indicating that 94.07, 93.83, and 97.94% match between the values of the predicted model and the values that were attained in the experimental data. Besides, the *r*^2^ values were comparable to adjusted *r*^2^, demonstrating good statistical model. The *p* values for the lack of fit were identified as 0.2462, 0.4015, and 0.5001, highlighting that the lack of fit of the models was significant at *p* > 0.05. Hence, the results indicate that the models could predict the TFC, TPC, and XO inhibitory activity of *E*. *hirta* extract efficiently when independent variables were within the ranges depicted here.

### 3.2. Effect of Extraction Parameters on Total Flavonoid Content (TFC)

**Figure 1:**
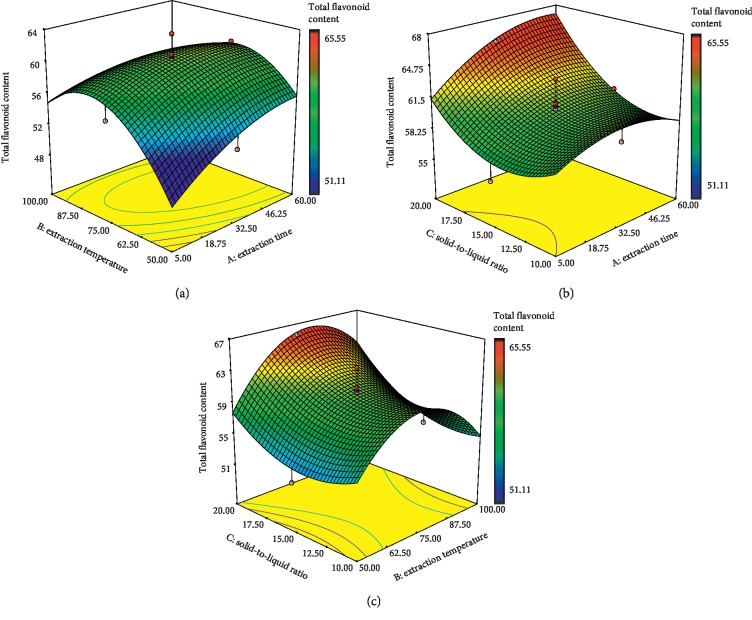
Response surface plots of *E*. *hirta* showing the effect of (a) extraction time and extraction temperature, (b) solid-to-liquid ratio and extraction time, (c) solid-to-liquid ratio and extraction temperature on TFC. Color gradients indicate the level of optimization (red = high, green = intermediate, and blue = low).

Flavonoids are the most common group of polyphenolic compounds in the human diet and are found ubiquitously in plants [25]. They are categorized mainly into flavone, flavanol, and flavanone compounds. [Supplementary-material supplementary-material-1] (Supplementary data) shows that all independent variables (extraction time, temperature, and solid-to-liquid ratio) had a significant effect on TFC of *E*. *hirta* extract. Among these, solid-to-liquid ratio (*X*_3_) was found to be the major effect on TFC in comparison with other variables, which may be due to the high *F*-value of 33.99. Interestingly, the analysis of the results also showed that all the interactions between time and temperature (*X*_1_*X*_2_), time and solid-to-liquid ratio (*X*_1_*X*_3_), and temperature and solid-to-liquid ratio (*X*_2_*X*_3_) were highly significant factors affecting the yield of TFC (*p* < 0.05). Based on the previous studies, extraction temperature, time, and solid-to-liquid ratio are considered to be the important parameters for the extraction efficiency of flavonoid compounds [26, 27]. Figures 1(a)–1(c) show the three-dimensional response surface plots for the influences of extraction temperature, time, and solid-to-liquid ratio on TFC. The response surface plots are very useful to see the interaction effects of the factors on the responses. As in Figure 1(a), the TFC extraction yields significantly increased as the extraction temperature and time increased from 50 to 75°C and 5 to 60 min, respectively, but started to decrease towards 100°C while keeping the solid-to-liquid ratio as constant. This was supported by Sheng et al. [28] where the yield of total flavonoids in the plant extract rose gradually with the increase of temperature from 50° to 90°C. The increase of extraction temperature promotes the solvent extraction process of the plant extract due to multiple effects of temperature on the mass transfer process by enhancing both diffusion coefficients and the solubility of flavonoid compounds as well as promoting the degradation of the plant matrix [26, 29].

Figures 1(b) and 1(c) display the effect of solid-to-liquid ratio-extraction time and solid-to-liquid ratio-extraction temperature on TFC of *E*. *hirta*, respectively. The yield of TFC increased when the solid-to-liquid ratio and extraction time increased (Figure 1(b)). As shown in Figure 1(c), as the ratio of solid-to-liquid increased from 1 : 15 to 1 : 20, the yield of TFC increased significantly from 59.82 mg/g to 64.06 mg/g with an increase in extraction temperature. This was supported by the previous study where the TFC of *Gynura medica* leaves significantly increased from 24.48 to 35.39 mg kaempferol/g DM as the liquid-to-solid ratio increased from 10 to 40 (v/w) [26]. The same trend was also observed by Sheng et al. [28] where the TFC increased gradually with the increase of liquid-to-solid ratio from 10 : 1 to 18 : 1. This might be due to the increase of the driving force for the mass transfer of the TFC, resulting in the high TFC obtained at the end of the study [26].

### 3.3. Effect of Extraction Parameters on Total Phenolic Content (TPC)

**Figure 2:**
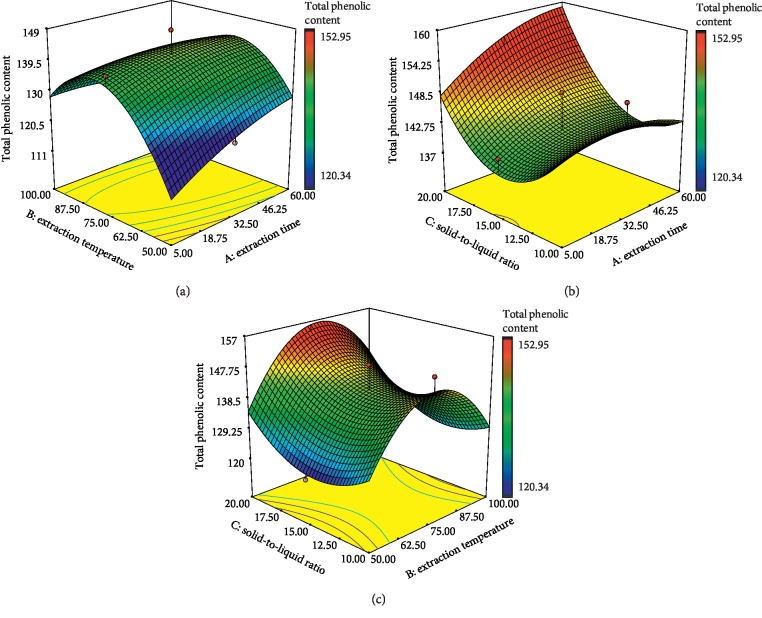
Response surface plots of *E*. *hirta* showing the effect of (a) extraction time and extraction temperature, (b) solid-to-liquid ratio and extraction time, and (c) solid-to-liquid ratio and extraction temperature on total phenolic contents. Color gradients indicate the level of optimization (red = high, green = intermediate, and blue = low).

One of the most important groups of secondary metabolites produced by all plants is phenolic compounds. Phenolic compounds are secondary metabolites that are derivatives of the pentose phosphate, shikimate, and phenylpropanoid pathways in plants [30]. Phenolics include simple phenols, phenolic acids (benzoic and cinnamic acid derivatives), coumarins, flavonoids, stilbenes, hydrolyzable and condensed tannins, lignans, and lignins, acting mainly as phytoalexins, attractants for pollinators, contributors to plant pigmentation, antioxidants, and protective agents against ultraviolet (UV) light [31]. [Supplementary-material supplementary-material-1] (Supplementary data) shows that all independent variables (extraction time, temperature, and solid-to-liquid ratio) had the significant effect on TPC of *E*. *hirta* extract. Among these, solid-to-liquid ratio (*X*_3_) was found to be the major effect on TPC in comparison with other variables, which may due to the high *F*-value of 19.42. Interestingly, the analysis of the results also showed that the interactions between time and temperature (*X*_1_*X*_2_) and time and solid-to-liquid ratio (*X*_1_*X*_3_) were highly significant factors affecting the yield of TFC (*p* < 0.05) except for temperature and solid-to-liquid ratio (*X*_2_*X*_3_). Figures 2(a)–2(c) show the response surface plots for the influences of extraction temperature, extraction time, and solid-to-liquid ratio on TPC. As shown in Figure 2(a), TPC was increased as the extraction temperature increased and reached the maximum at 75°C but started to decrease towards 100°C especially at high levels of extraction time, implying that beyond a certain value of temperature, phenolic compounds can be denatured [32, 33]. Previous study has demonstrated that the yield of phenolic compounds was increased significantly over the extraction temperature range (25–75°C) where the maximum of TPC was at 65°C [34]. In fact, heat has been found to enhance the recovery of phenolic compounds [14, 35]. It was believed that high temperature increased and enhanced the diffusion and solubility rate of phenolic compounds by softening the plant tissue as well as disrupting the interactions between the phenolic compounds and protein or polysaccharides [36]. It is interesting to note that in the present study, increasing extraction temperature had a positive effect to TPC. From this scenario, it is believed that the phenolic compounds present in *E*. *hirta* are thermally stable up to 75°C.  Figure 2(b) shows that the TPC increased significantly as the solid-to-liquid ratio increased from 1 : 15 to 1 : 20 with the increase of extraction time.  Figure 2(c) also shows that the TPC increased significantly as the solid-to-liquid ratio increased from 1 : 15 to 1 : 20 with the increase of extraction temperature up to 75°C. Hence, from these figures, the solid-to-liquid ratio could drastically affect the yield of phenolic compounds during the extraction process. The similar result was observed from the previous study where the yield of phenolic compounds from date seeds water extract increased from 2.5 to 5.5 g/100 g as solvent ratio increased to the 60 : 1 level [37]. The same results were reported for the milled berries extraction where higher solid-to-liquid ratio resulted in higher yield of phenolic compounds [38]. This was in line with the mass transfer principle [39].

### 3.4. Effect of Extraction Parameters on XO Inhibitory Activity

**Figure 3:**
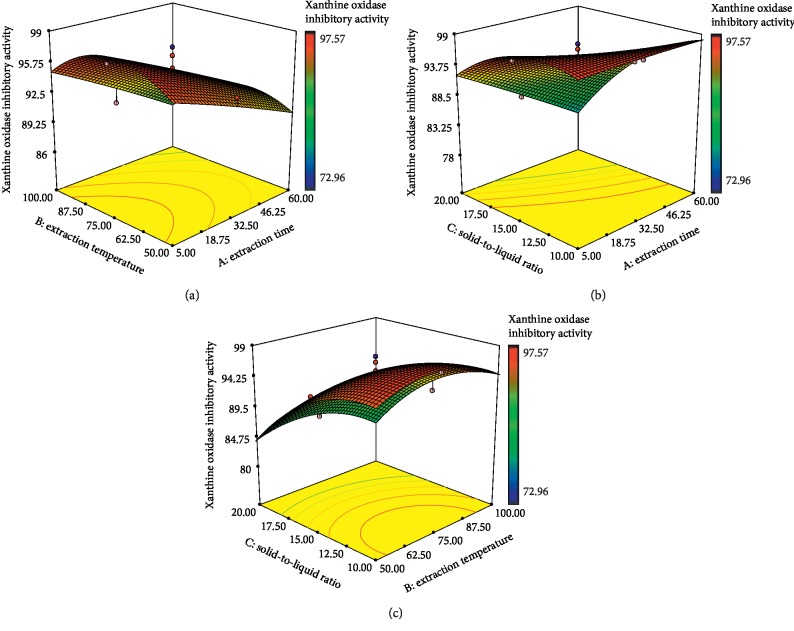
Response surface plots of *E*. *hirta* showing the effect of (a) extraction time and extraction temperature, (b) solid-to-liquid ratio and extraction time, and (c) solid-to-liquid ratio and extraction temperature on xanthine oxidase inhibitory activity. Color gradients indicate the level of optimization (red = high, green = intermediate, and blue = low).

XO inhibition assay is a common assay used for determining the anti-gout activity of the plant extracts [40]. XO catalyses the oxidation of hypoxanthine to xanthine and then to uric acid, which plays a crucial role in gout [41]. As reviewed by Ling and Bochu [42], phenolic and flavonoid compounds have been reported to be the potent plant-based XO inhibitors which make them crucial as anti-gout agents. Few studies from the previous years have successfully optimized the extraction conditions for higher XO inhibitory activity of the plants [43–45]. Figures 3(a)–3(c) illustrate the response surface plots for the influences of extraction temperature, extraction time, and solid-to-liquid ratio on XO inhibitory activity. [Supplementary-material supplementary-material-1] (Supplementary data) shows that all independent variables (extraction time, temperature, and solid-to-liquid ratio) had the significant effect on XO inhibitory activity of *E*. *hirta* extract. Among these, extraction time (*X*_1_) and solid-to-liquid ratio (*X*_3_) were found to be the major effects on XO inhibitory activity in comparison with extraction temperature (*X*_2_), which may due to the high *F*-values of 66.27 and 239.46, respectively. Interestingly, the analysis of the results also showed that the interactions between time and solid-to-liquid ratio (*X*_1_*X*_3_) was highly significant factor affecting the XO inhibitory activity (*p* < 0.05). As shown in Figure 3(a), XO inhibitory activity increased when the temperature increased from 50 to 75°C and started to decrease towards 100°C as the extraction time decreased. This was in agreement with Edirs et al. [46] where the antidiabetic activity of Kursi Wufarikun Ziyabit using protein tyrosine phosphatase-1B and *α*-glucosidase inhibition assays increased as the extraction temperature increased from 70 to 75°C and 65 to 70°C, respectively. This explains that an increase in the extraction temperature beyond certain value will denature or decompose some of the valuable compounds responsible for inhibiting XO activity [47]. In fact, the high temperature may cause the enzymes to be denatured as they are heat sensitive [48].

Meanwhile, Figure 3(b) clearly shows that the XO inhibitory activity decreased when the extraction time and solvent-to-liquid ratio increased. The XO inhibitory activity reached at maximum level (95.37%) when the extraction time and solvent to liquid ratio were 32.5 min and 1 : 15 g/ml, respectively. This implied that excessive extraction time (more than 32.5 min) was no longer useful to extract more active compounds from *E*. *hirta* which contributed to antigout activity. In fact, oxidation of the active compounds during the prolonged time of extraction process could also reduce the XO activity. However, lesser contact time during the extraction process of the plants would not allow good mixing between the samples and solvent, resulting in low XO inhibitory activity [43]. In addition, the interaction effects between the extraction temperature and solid-to-liquid ratio on XO inhibitory activity with a constant value of the extraction time at 32.5 min are shown in Figure 3(c). It can be seen that solid-to-liquid ratio gave greater impact on the XO inhibitory activity of *E*. *hirta* regardless of the temperature used. This was in agreement with Mehmood et al. [49] who also showed that the solute solvent ratio gave a significant effect on XO inhibition.

### 3.5. Optimization of Extraction Conditions

In this study, the desirability function approach suggested that the optimal extraction conditions for *E*. *hirta* water extract in obtaining maximum XO inhibitory activity (91.42%), TFC (67.56 mg RE/g), and TPC (155.21 mg GAE/g) were at 79.07°C with 17.42 min and 1 : 20 of solid-to-liquid ratio. The predicted values of all the responses were close to the experimental values with less than 10% error, which is relatively desirable and all the experimental data were within ±95% prediction intervals. Besides, the accuracy of our model was confirmed as no significant difference was observed (*p* > 0.05) between the experimental values and the predicted values (Table 3). Therefore, it can be concluded that the model from CCD was accurate and reliable enough for predicting the TFC and TPC as well as anti-gout activity of *E*. *hirta*.

### 3.6. Liquid Chromatography-Mass Spectrometry (LC-MS/MS) Metabolite Profile of *E*. *hirta* Extract

**Figure 4:**
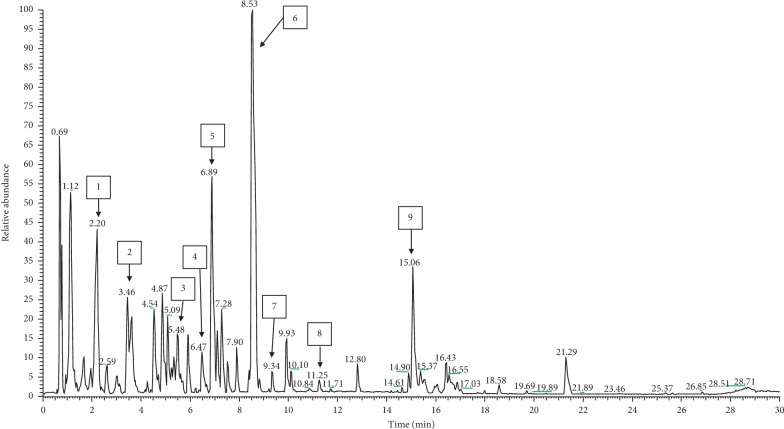
LC-MS chromatogram, acquired in the negative ion mode, from an extract of *E*. *hirta* where peak labelling represents the compounds identified and listed in Table 4.

The *E*. *hirta* optimized extracts were analyzed and profiled by LC-MS/MS analysis in positive and negative ionization modes in order to perform a qualitative characterization of chemical constituents in *E*. *hirta*. To our knowledge, this is the first validated method on the detection of active compounds in *E*. *hirta* whole plant excluding roots using LC-MS/MS analysis. The base peak chromatogram is depicted in Figures 4 (negative ionization mode) and 5 (positive ionization mode). Compounds were characterized for their retention times, the absorption spectrum in the UV-vis region, the mass spectrum obtained by MS-ESI, and the fragmentation profile as well as compared with the previous literature reports [50–53]. The results obtained from the LC-MS/MS analysis allowed the tentative assignment of nine chemical constituents using negative ionization mode comprising four phenolic acids: neochlorogenic acid ([M − H]^−^ ion at *m*/*z* 353), caffeic acid ([M − H]^−^ ion at *m*/*z* 179.034), syringic acid ([M − H]^−^ ion at *m*/*z* 197.045), and ellagic acid ([M − H]^−^ ion at *m*/*z* 300.999) and four flavonoids: quercetin-3*β*-D-glucoside ([M − H]^−^ ion at *m*/*z* 463.087), astragalin ([M − H]^−^ ion at *m*/*z* 447.094), afzelin ([M + FA − H]^−^ ion at *m*/*z* 477.104), quercetin ([M − H]^−^ ion at *m*/*z* 301.035) as well as one fatty acid: corchorifatty acid F ([M − H]^−^ ion at *m*/*z* 346.081). In addition, LC-MS/MS analysis also allowed the tentative assignment of two chemical constituents using positive ionization mode: 2-undecanone 2,4-dinitrophenylhydrazone ([M + H]^+^ ion at *m*/*z* 351.4) and demexiptiline ([M + H]^+^ ion at *m*/*z* 279.3). In this study, the negative mode ESI was more sensitive for the detection of the phenolics and flavonoids in the extract. The same observations were also shown by Zakaria et al. [24] and Keskes et al. [54].

Previous studies have demonstrated that flavonoids and phenolic acids possess high biological and pharmacological activities [55]. In the LC chromatogram, the peak at retention time (*R*_T_) 2.20 min was assigned to the presence of neochlorogenic acid which was also reported by the previous studies [56, 57]. In fact, neochlorogenic acid showed potent anti-inflammatory activity [58]. In this study, astragalin (kaempferol-3-O-glucoside), a natural flavonoid, was the main compound obtained in *E*. *hirta* water extract. Previous study also reported the presence of astragalin in other *Euphorbia* species [59]. As reviewed by Riaz et al. [60], astragalin showed potent anti-inflammatory activity by inhibiting the production of inflammatory mediators such as interleukin-1 beta (IL-1*β*), interleukin-6 (IL-6), and tumor necrosis factor-alpha (TNF-*α*) in macrophages [61, 62]. Moreover, Jia et al. [63] demonstrated for the first time that astragalin effectively inhibited the worsening of synovial inflammation and joint destruction in the animal model. In fact, flavonoid compounds from Malaysian medicinal plants showed potent anti-gout and anti-inflammatory activities from the previous studies [64, 65]. Hence, in this present study, we speculated that the anti-gout effects of *E*. *hirta* might be due to the presence of high concentration of astragalin in the extract.

## 4. Conclusion

RSM was successfully employed to optimize the phytochemical compounds and anti-gout activity of *E*. *hirta* where CCD proved to be an efficient tool for the optimization of these parameters. The second-order polynomial models for predicting responses were obtained, and the best combination of extraction temperature, time, and solid-to-liquid ratio were found to be 79.07°C with 17.42 min and 1 : 20, respectively. Further investigation on the secondary metabolites present in the optimized extract of *E*. *hirta* using LC-MS analysis revealed the presence of neochlorogenic acid, quercetin-3*β*-D-glucoside, syringic acid, caffeic acid, ellagic acid, astragalin, afzelin, quercetin, corchorifatty acid F, 2-undecanone 2,4-dinitrophenylhydrazone, and demexiptiline. Hence, *E*. *hirta* could be a natural source of polyphenols compounds which serve as ingredients of functional foods and be used in pharmaceuticals for the treatment of gout disease.

## Figures and Tables

**Figure 5:**
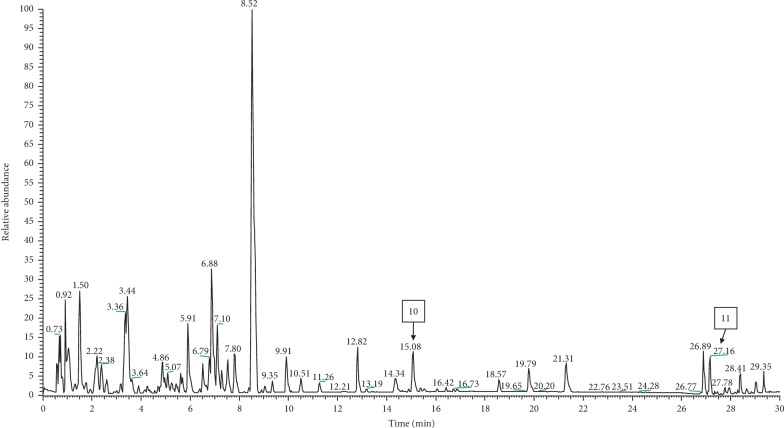
LC-MS chromatogram, acquired in the positive ion mode, from an extract of *E*. *hirta* where peak labelling represents the compounds identified and listed in Table 4.

**Table 1:** Experimental design for the extraction process from central composite design.

Run order	*X*_1_ (extraction time) (min)	*X*_2_ (extraction temperature) (°C)	*X*_3_ (solid-to-liquid ratio) (g/ml)
1^a^	32.5	75	15
2^a^	32.5	75	15
3^a^	32.5	75	15
4	32.5	50	15
5^a^	32.5	75	15
6	32.5	75	20
7^a^	32.5	75	15
8	5	50	20
9	32.5	75	10
10	60	50	10
11	60	50	20
12	60	100	10
13	60	75	15
14	32.5	100	15
15	5	100	20
16	5	75	15
17	5	100	10
18	5	50	10
19	60	100	20
20^a^	32.5	75	15

^a^Center point.

**Table 2:** Response surface central composite design (uncoded) and results for total flavonoid content, total phenolic content, and xanthine oxidase inhibitory activity.

Run order	*X*_1_ (extraction time) (min)	*X*_2_ (extraction temperature) (°C)	*X*_3_ (solid-to-liquid ratio) (g/ml)	*Y*_1_/total flavonoid content (mg RE/g)^b^	*Y*_2_ (total phenolic content (mg GAE/g)^c^	*Y*_3_ (xanthine oxidase inhibitory activity) (%)
1^a^	32.5	75	15	50.92	130.53	95.13
2^a^	32.5	75	15	60.62	140.76	97.66
3^a^	32.5	75	15	60.54	140.63	97.28
4	32.5	50	15	50.78	120.76	90.07
5^a^	32.5	75	15	60.38	140.28	96.60
6	32.5	75	20	60.55	150.95	84.95
7^a^	32.5	75	15	60.44	130.66	96.44
8	5	50	20	60.34	150.87	89.75
9	32.5	75	10	50.27	120.49	94.57
10	60	50	10	40.98	110.38	95.31
11	60	50	20	60.99	150.97	73.32
12	60	100	10	50.11	110.69	94.94
13	60	75	15	50.44	120.43	87.39
14	32.5	100	15	50.89	130.45	89.26
15	5	100	20	70.41	160.92	88.93
16	5	75	15	60.26	140.30	97.44
17	5	100	10	50.17	110.81	94.95
18	5	50	10	40.96	110.34	94.83
19	60	100	20	60.75	150.42	72.96
20^a^	32.5	75	15	60.42	140.66	94.19

^a^Center point. ^b^Total flavonoid content was expressed as mg rutin equivalents in 1 g of dried sample (mg RE/g). ^c^Total phenolic content was expressed as mg gallic acid equivalents in 1 g of dried sample (mg GAE/g).

**Table 3:** Differences between the predicted value and the experimental value.

Parameters	Predicted value	Experimental value	Percentage of error (%)
Total flavonoid content (mg RE/g)	64.31 ± 0.00^a^	67.56 ± 0.83^a^	4.81
Total phenolic content (mg GAE/g)	152.95 ± 0.00^b^	155.21 ± 0.67^b^	1.46
Xanthine oxidase inhibitory activity (%)	88.78 ± 0.00^c^	91.42 ± 0.74^c^	2.89

Values were presented as mean ± standard deviation. Same superscripts within the row indicate no significant difference (*p* > 0.05) between predicted and experimental values.

**Table 4:** Tentative identification of chemical constituents in the *E*. *hirta* water extract.

No	Tentative assignment	*R*_T_ (min)	Molecular weight	Precursor type	Precursor *m*/*z*	Molecular formula	MS/MS fragment ions	References
1	Neochlorogenic acid	2.2	354.09	[M − H]^−^	353	C_16_ H_18_ O_9_	191, 179	Massbank: PM013904
2	Caffeic acid	3.46	180.04	[M − H]^−^	179.0341	C_9_ H_8_ O_4_	135, 179, 134	Massbank: FiehnHILIC001102
3	Syringic acid	5.48	198.052	[M − H]^−^	197.045	C_9_ H_10_ O_5_	182, 166, 197	Massbank: FiehnHILIC001522
4	Ellagic acid	6.47	302.006	[M − H]^−^	300.999	C_14_ H_6_ O_8_	303, 300	Massbank: FiehnHILIC001170
5	Quercetin-3*β*-D-glucoside	6.89	464.09	[M − H]^−^	463.0879	C_21_ H_20_ O_12_	300, 301, 271	Pubchem (CID5280804)
6	Astragalin	8.53	448.1	[M − H]^−^	447.094	C_21_ H_20_ O_11_	447, 285, 284	Massbank: CCMSLIB00000845312
7	Afzelin	9.34	432.1	[M + FA − H]^−^	477.104	C_21_ H_20_ O_10_	285, 431, 284	Massbank: CCMSLIB00000845703
8	Quercetin	11.25	302.04	[M − H]^−^	301.0359	C_15_ H_10_ O_7_	107, 121, 151, 178, 301	Massbank: FiehnHILIC002955
9	Corchorifatty acid F	15.06	328.224	[M − H]^−^	346.081	C_18_ H_32_ O_5_	235, 311, 219	Pubchem (CID44559173)
10	2-Undecanone 2,4-dinitrophenylhydrazone	15.08	350.4	[M + H]^+^	351.4	C_17_ H_26_ N_4_ O_4_	351, 352	Pubchem (CID 5717665)
11	Demexiptiline	27.16	278.3	[M + H]^+^	279.3	C_18_ H_18_ N_2_ O	204, 203, 149	Massbank: WA002119

## Data Availability

The datasets generated and/or analyzed during the current study are available from the first author on reasonable request.

## Abbreviations

CCD: Central composite design
LC-MS: Liquid chromatography-mass spectrometry
RSM: Response surface methodology
TFC: Total flavonoid content
TPC: Total phenolic content
XO: Xanthine oxidase.

## Supplementary Materials

Supplementary Materials Table S1: the regression coefficients and results of ANOVA for response surface quadratic model of total flavonoid content. Table S2: the regression coefficients and results of ANOVA for response surface quadratic model of total phenolic content. Table S3: the regression coefficients and results of ANOVA for xanthine oxidase inhibitory activity.
