# Supplementary material for: Optimization of Extraction Conditions of Phytochemical Compounds and Anti-Gout Activity of Euphorbia hirta L. (Ara Tanah) Using Response Surface Methodology and Liquid Chromatography-Mass Spectrometry (LC-MS) Analysis
Source: Evid Based Complement Alternat Med. 2020 Jan 24;2020:4501261. doi: 10.1155/2020/4501261 (PMC7007754; doi:10.1155/2020/4501261)
Supplement: Supplementary Materials — Table S1: the regression coefficients and results of ANOVA for response surface quadratic model of total flavonoid content. Table S2: the regression coefficients and results of ANOVA for response surface quadratic model of total phenolic content. Table S3: the regression coefficients and results of ANOVA for xanthine oxidase inhibitory activity. [file 4501261.f1.docx]

**Table S1:** The regression coefficients and results of ANOVA for response surface quadratic model of total flavonoid content

| **Source** | **Sum of squares** | **Df** | **Mean square** | ***F* value** | ***P* value** |
| --- | --- | --- | --- | --- | --- |
| Model | 324.93 | 9 | 36.10 | 17.63 | <0.0001 |
| *X_1_* | 23.59 | 1 | 23.59 | 11.52 | 0.0068 |
| *X_2_* | 12.21 | 1 | 12.21 | 5.96 | 0.0347 |
| *X_3_* | 69.59 | 1 | 69.59 | 33.99 | 0.0002 |
| *X_1_X_2_* | 41.50 | 1 | 41.50 | 20.27 | 0.0011 |
| *X_1_X_3_* | 13.16 | 1 | 13.16 | 6.43 | 0.0296 |
| *X_2_X_3_* | 13.16 | 1 | 13.16 | 6.43 | 0.0296 |
| *X_1_^2^* | 8.14 | 1 | 8.14 | 3.97 | 0.0742 |
| *X_2_^2^* | 90.45 | 1 | 90.45 | 44.18 | <0.0001 |
| *X_3_^2^* | 22.18 | 1 | 22.18 | 10.83 | 0.0081 |
| *R^2^* | 0.9407 |  |  |  |  |
| Residual | 20.47 | 10 | 2.05 |  |  |
| Lack of Fit | 13.45 | 5 | 2.69 | 1.92 | 0.2462 |
| Pure Error | 7.02 | 5 | 1.40 |  |  |
| Cor Total | 345.40 | 19 |  |  |  |

**Table S2:** The regression coefficients and results of ANOVA for response surface quadratic model of total phenolic content

| **Source** | **Sum of squares** | **Df** | **Mean square** | ***F* value** | ***P* value** |
| --- | --- | --- | --- | --- | --- |
| Model | 1835.54 | 9 | 203.95 | 16.89 | <0.0001 |
| *X_1_* | 60.76 | 1 | 60.76 | 5.03 | 0.0487 |
| *X_2_* | 62.35 | 1 | 62.35 | 5.16 | 0.0464 |
| *X_3_* | 234.45 | 1 | 234.45 | 19.42 | 0.0013 |
| *X_1_X_2_* | 261.75 | 1 | 261.75 | 21.68 | 0.0009 |
| *X_1_X_3_* | 76.14 | 1 | 76.14 | 6.31 | 0.0308 |
| *X_2_X_3_* | 23.53 | 1 | 23.53 | 1.95 | 0.1929 |
| *X_1_^2^* | 16.88 | 1 | 16.88 | 1.40 | 0.2643 |
| *X_2_^2^* | 817.13 | 1 | 817.13 | 67.69 | <0.0001 |
| *X_3_^2^* | 216.72 | 1 | 216.72 | 17.95 | 0.0017 |
| *R^2^* | 0.9383 |  |  |  |  |
| Residual | 120.72 | 10 | 12.07 |  |  |
| Lack of Fit | 67.41 | 5 | 13.48 | 1.26 | 0.4015 |
| Pure Error | 53.31 | 5 | 10.66 |  |  |
| Cor Total | 1956.26 | 19 |  |  |  |

**Table S3:** The regression coefficients and results of ANOVA for xanthine oxidase inhibitory activity

| **Source** | **Sum of squares** | **Df** | **Mean square** | ***F* value** | ***P* value** |
| --- | --- | --- | --- | --- | --- |
| Model | 780.65 | 9 | 86.74 | 52.85 | < 0.0001 |
| *X_1_* | 108.77 | 1 | 108.77 | 66.27 | < 0.0001 |
| *X_2_* | 12.63 | 1 | 12.63 | 7.70 | 0.0196 |
| *X_3_* | 393.00 | 1 | 393.00 | 239.46 | < 0.0001 |
| *X_1_X_2_* | 3.16 | 1 | 3.16 | 1.93 | 0.1952 |
| *X_1_X_3_* | 97.09 | 1 | 97.09 | 59.16 | < 0.0001 |
| *X_2_X_3_* | 4.40 | 1 | 4.40 | 2.68 | 0.1328 |
| *X_1_^2^* | 0.072 | 1 | 0.072 | 0.044 | 0.8378 |
| *X_2_^2^* | 23.32 | 1 | 23.32 | 14.21 | 0.0037 |
| *X_3_^2^* | 30.26 | 1 | 30.26 | 18.44 | 0.0016 |
| *R^2^* | 0.9794 |  |  |  |  |
| Residual | 38.44 | 10 | 1.64 |  |  |
| Lack of Fit | 29.74 | 5 | 1.64 | 1.00 | 0.5001 |
| Pure Error | 8.70 | 5 | 1.64 |  |  |
| Cor Total | 1003.07 | 19 |  |  |  |
